# Research on robot positioning error compensation algorithm based on the Dog Leg and PSONN algorithm

**DOI:** 10.1371/journal.pone.0331136

**Published:** 2025-09-16

**Authors:** Ming Li, Rongsheng Lu

**Affiliations:** School of Instrument Science and Opto-electronic Engineering, Hefei University of Technology, Hefei, China; Beijing Institute of Technology, CHINA

## Abstract

The absolute positioning accuracy of industrial robots is much lower than that of repetitive. In this paper, an error compensation algorithm for industrial robots is proposed, which included the kinematic parameter calibration based on the enhanced Dog Leg algorithm, the odd point error prediction based on the Particle Swarm Optimization Neural Network (PSONN) algorithm, and the positioning error calculation based on the Spatial Grid Multipoint Interpolation (SGMI) algorithm. The proposed algorithm reduce the robot localization error in three progressive steps, which combines the interpretability of traditional algorithms and the nonlinear effect of neural networks, avoiding the low accuracy of traditional algorithms and the local optimal phenomenon of neural networks. The robot end positioning error model developed in this paper, mainly includes kinematic parameters, return angle, deceleration ratio relative error coefficients, joint angle coupling coefficients, and base coordinate system error. The experimental results demonstrate that, after calibrating kinematic parameter, the positioning error is reduced from 3.158 mm to 0.406 mm, the uncertainty is reduced from 1.726 mm to 0.160 mm. After compensating by the SGMI algorithm, the positioning error is reduced from 0.406 mm to 0.0685 mm. The results also demonstrate that the proposed SGMI algorithm calibrate the kinematic parameter effectively and reduced the positioning error of the industrial robot significantly.

## 1. Introduction

The industrial robot is very important automation equipment in the manufacturing industry, which integrates the technology of machinery, electronics, sensors, control and many other fields. The industrial robot is widely used in handling device industries, welding industries military manufacturing industries and other industries. In the current, the repetitive positioning error is generally approximately 0.2 mm to 1 mm, but the absolute positioning error is bigger than the repetitive, reaching approximately 1 mm to 3 mm. Improving the absolute positioning accuracy can be done by calibrating the kinematic parameters. The kinematic parameters calibration process is generally divided into four steps, which included modeling, measurement, calibration and compensation [1].In recent decades, researchers have conducted studies on calibration techniques to industrial robots.

In terms of traditional algorithms, Luo et al [2] combined the LM (Levenberg Marquard) algorithm and the differential evolution algorithms to calibrate industrial robots. Fan et al [3] used the kinematic parameter errors calibrated by the LM algorithm, as the central value of the initial individual of the BAS algorithm. They reduce the positioning error of the UR5 from 0.7332 mm to 0.1392 mm. Cao et al [4] used the Extended Kalman Filter (EKF) algorithms and the Dual Quantum Behaviour Particle Swarm Optimization algorithms, to calibrate industrial robots.

In terms of bionic algorithms, Chen et al [5] enhanced the Beetle Antennae Search (BAS) algorithm to calibrate industrial robot. Shi et al [6] used Particle Swarm Optimization (PSO) algorithm to solve kinematic parameter errors iteratively. They optimized the PSO algorithm, using the sine strategy, cosine strategy and the trust domain algorithm. After calibration, the average distance error was enhanced from 1.1601 mm to 0.2260 mm. The accuracy was enhanced by 80.52%. The standard deviation was reduced from 0.6582 mm to 0.1412 mm.

In terms of the artificial neural network (ANN) algorithms, there are also many researchers and corresponding results. Neural Network is an emerging technology, developed in recent years, which is also used to solve the kinematic parameters. Wang et al [7] compensatory target position error, using neural network model. Li et al [8] predict and compensate the robot error, using the PSO algorithm. Jan et al [9] identify geometric errors and non-geometric errors of DR06, using the Neural Network algorithms with radial basis function. The positioning error was improved from approximately 5.8 mm to 1.8 mm. Nguyen et al [10] identifies PUMA’s geometric parameters using an EKF algorithm, and identifies its non-geometric error sources.

In terms of error similarity, Zhou et al [11] proposed the concept of spatial similarity. Zhou et al proposed an iterative optimization and neural network compensation, based semi parametric kinetic model identification method. Cai et al [12] found that modeling the industrial robot error, based on the kriging method and the concept of error similarity, can effectively improve the compensation accuracy. Gao et al [13,14] recognize the kinematic parameters, using the extended Kalman filter algorithm, and established error compensation database based on grid. They predicted the joint angle errors, using the joint angle distance as the weight and the error compensation database.

In terms of calibration tools, Zhao et al [15] used laser tracker to measure the end position to calibrate industrial robot. Wang et al [7] used neural network model to compensate for the target positional error.

Research in robot localization error algorithms has focused on traditional algorithms (e.g., EKF, BAS, etc.) in and nonlinear algorithms (e.g., neural networks). Although the above studies of neural networks effectively improve the robot positioning accuracy, the number of the model parameters is less. The solution usually falls into local optimization, using neural network algorithm. Aiming at the above problems, we proposed a new comprehensive compensation algorithm. This algorithm avoids both the disadvantage of low accuracy of traditional algorithms and the situation where nonlinear algorithms fall into local optimality. However, the proposed algorithm also has some limitations, it can’t be realized for real-time. The proposed algorithm consists of the following steps. Firstly, the kinematic parameters and other parameters of positioning error are calibrated, using the enhanced Dog Leg algorithm. Secondly, the PSO algorithms was used to optimize the initial weights and the thresholds of the neural network. We predicted the positioning error of odd points in the robot workspace, using the PSONN algorithm. Thirdly, based on the principle of spatial similarity, we predicted the positioning errors, using the SGMI algorithm. Fourthly, to verify the effectiveness of the PSONN algorithm, proposed in this paper, we conducted an experimental on FANUC robot with six degrees of freedom.

This paper is divided into 5 sections. In section 2, the kinematics model and the positioning error model are investigated. In section 3, the error comprehensive compensation algorithms are investigated, mainly including the improvement Dog Leg algorithm, the PSONN algorithm, and the SGMI algorithm. In section 4, experiments were performed on FANUC LR MATE robot. In section 5, we summarize the conclusions of this paper.

## 2. Robot error modeling

### 2.1 Kinematic modeling

The main kinematic modeling methods currently used in robots include the Denavit Hartenberg (DH)model [16]、the Modified DH model (MDH) [17]、the S model [18], the Complete and Parametrically Continuous (CPC) model [19], the Zero Baseline model, and the Product of Exponentials (POE) model [20].

The DH model is currently the most widely used method for modeling robot kinematics. The DH model is divided into the Stand DH model and the MDH model, i.e., the pre-link coordinate system and the post- link coordinate system.

When the two neighboring joints are parallel, the DH model is odd. To solve the odd problem of the DH model, the MDH model emerged. The MDH model is based on the DH model, and an addition angle $\beta_{i}$ is increasing in the Y-axis to solve the odd problem. In this robot, the 2nd joint and the 3rd joint are parallel. To solve this odd problem, let $d_{3}$ =0, and add a micro rotate angle $\beta_{3}$.

**Fig 1:**
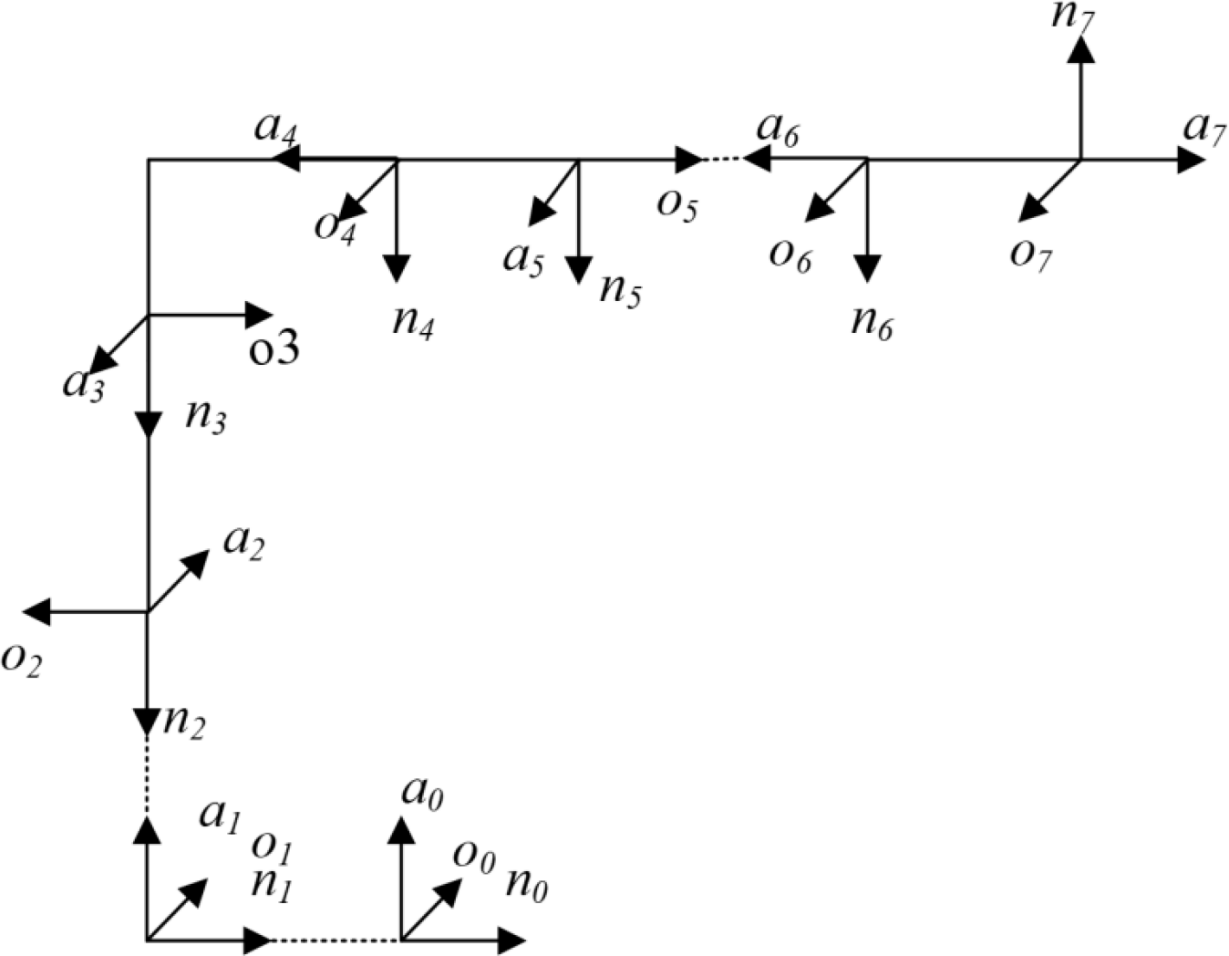
The coordinate system of FANUC robot. $\left(n_{0},o_{0},a_{0}\right)$ indicate the direction of the Base coordinate system of the FANUC robot; $\left(n_{1},o_{1},a_{1}\right)$ indicate the direction of the 1st joint coordinate system; $\left(n_{2},o_{2},a_{2}\right)$ indicate the direction of the 2nd joint coordinate system; $\left(n_{3},o_{3},a_{3}\right)$ indicate the direction of the 3rd joint coordinate system; $\left(n_{4},o_{4},a_{4}\right)$ indicate the direction of the 4th joint coordinate system; $\left(n_{5},o_{5},a_{5}\right)$ indicate the direction of the 5th joint coordinate system; $\left(n_{6},o_{6},a_{6}\right)$ indicate the direction of the 6th joint coordinate system; $\left(n_{7},o_{7},a_{7}\right)$ indicate the direction of the SMR coordinate system.


The MDH model in this paper, need 5 steps, establishes the transformation relationship from the (i-1)th joint coordinate system to the ith joint coordinate system, in that order: 1) rotate $\alpha_{i-1}$ around the axis $X_{i-1}$, so that the axis $Z_{i-1}$ is parallel to the axis $Z_{i}$; 2) move $a_{i-1}$ along the axis $X_{i-1}$, so that the axis $Z_{i-1}$ is colinear with the axis $Z_{i}$; 3) rotate $\theta_{i}$ around the axis $Z_{i}$, so that the axis $X_{i-1}$ is parallel to the axis $X_{i}$; 4) move $d_{i}$ along the axis $Z_{i}$, so that the axis $X_{i-1}$ is colinear with the axis $X_{i}$ [16]; and 5) rotate $\beta_{i}$ around the axis $Y_{i}$. According to the above process, the neighboring joint coordinate system transformation relationship can be expressed as Eq. (1) [21]. In Eq. (1), ‘c’ stands for cos and ‘s’ stands for sin. Based on the MDH model, the coordinate system in Fig 1 was established, and the initial values of the kinematic parameters were also established, based on the robot’s factory parameters, as shown in Table 1.

**Table 1:** Nominal values of robot kinematic parameters.

Joint	$\alpha_{i-1}$ /°	$a_{i-1}$ /mm	$\theta_{i}^{0}$ /°	$d_{i}$ /mm	$\beta_{i}$ /°
1	0	0	0	0	—
2	−90	0	90	0	—
3	180	−260	0	—	0
4	90	−20	0	−290	—
5	−90	0	0	0	—
6	90	0	0	−70	—

$$T_{i}^{i-1}=Rot(X_{i-1},\alpha_{i-1})Trans(X_{i-1},a_{i-1})Rot(Z_{i},\theta_{i})Trans(Z_{i},d_{i})Rot(Y_{i},\beta_{i})$$

$$=\left[\begin{array}{cccc} {}*20cc\mathrm{\backslash nolimits}\theta_{i}c\mathrm{\backslash nolimits}\beta_{i} & -s\mathrm{\backslash nolimits}\theta_{i} & c\mathrm{\backslash nolimits}\theta_{i}s\mathrm{\backslash nolimits}\beta_{i} & a_{i-1}\\ s\mathrm{\backslash nolimits}\theta_{i}c\mathrm{\backslash nolimits}\alpha_{i-1}c\mathrm{\backslash nolimits}\beta_{i}+s\mathrm{\backslash nolimits}\alpha_{i-1}s\mathrm{\backslash nolimits}\beta_{i} & c\mathrm{\backslash nolimits}\theta_{i}c\mathrm{\backslash nolimits}\alpha_{i-1} & s\mathrm{\backslash nolimits}\theta_{i}c\mathrm{\backslash nolimits}\alpha_{i-1}s\mathrm{\backslash nolimits}\beta_{i}-s\mathrm{\backslash nolimits}\alpha_{i-1}c\mathrm{\backslash nolimits}\beta_{i} & -d_{i}s\mathrm{\backslash nolimits}\alpha_{i-1}\\ s\mathrm{\backslash nolimits}\theta_{i}s\mathrm{\backslash nolimits}\alpha_{i-1}c\mathrm{\backslash nolimits}\beta_{i}-c\mathrm{\backslash nolimits}\alpha_{i-1}s\mathrm{\backslash nolimits}\beta_{i} & c\mathrm{\backslash nolimits}\theta_{i}s\mathrm{\backslash nolimits}\alpha_{i-1} & s\mathrm{\backslash nolimits}\theta_{i}s\mathrm{\backslash nolimits}\alpha_{i-1}s\mathrm{\backslash nolimits}\beta_{i}+c\mathrm{\backslash nolimits}\alpha_{i-1}c\mathrm{\backslash nolimits}\beta_{i} & d_{i}c\mathrm{\backslash nolimits}\alpha_{i-1}\\ 0 & 0 & 0 & 1 \end{array}\right]$$ (1)

The transformation matrix $T_{7}^{0}$, from base to end coordinate system, is obtain through $T_{7}^{0}=T_{1}^{0}T_{2}^{1}T_{3}^{2}RT_{4}^{3}T_{5}^{4}T_{6}^{5}T_{7}^{6}$. The positional relationship of the end center in the base coordinate system, can be expressed as Eq. (2).


$$T_{7}^{0}=\left[\begin{array}{cccc} {}*20cn_{x} & o_{x} & a_{x} & p_{x}\\ n_{y} & o_{y} & a_{y} & p_{y}\\ n_{z} & o_{z} & a_{z} & p_{z}\\ 0 & 0 & 0 & 1 \end{array}\right]$$ (2)

Where, $p_{x},p_{y},p_{z}$ indicates the coordinate of the end coordinate system in the base coordinate system. The coordinates $P_{7}^{0}$ of the SMR in the base coordinate system are expressed as ($T_{1}^{0}\left(1,4\right)$, $T_{7}^{0}\left(2,4\right)$, $T_{7}^{0}\left(3,4\right)$); In Eq. (3), the error function is constructed as the distance between $P_{7}^{0}$ and the SMR coordinates measured by the laser tracker.


$$e\left(x\right)=\sqrt{{\left(T_{7}^{0}\left(1,4\right)-P_{7,x}^{0,M}\right)}^{2}+{\left(T_{7}^{0}\left(2,4\right)-P_{7,y}^{0,M}\right)}^{2}+{\left(T_{7}^{0}\left(3,4\right)-P_{7,z}^{0,M}\right)}^{2}}$$ (3)

### 2.2 Robot error modeling

In the process of manufacturing and assembling of robot, link length errors and joint angle errors are generated. This can lead to deviations between the robot’s kinematic parameters and the factory’s kinematic parameters. This piece of deviation, which named the MDH parameter error, can be expressed as Eq. (4).


$$\Delta P_{g}=\frac{\partial P}{\partial \alpha_{i-1}}\Delta \alpha_{i-1}+\frac{\partial P}{\partial a_{i-1}}\Delta a_{i-1}+\frac{\partial P}{\partial \theta_{i}^{0}}\Delta \theta_{i}^{0}+\frac{\partial P}{\partial d_{i}}\Delta d_{i}+\frac{\partial P}{\partial \beta_{i}}\Delta \beta_{i}$$ (4)

The joint angle $\theta_{i}$ in Eq. (1), was consist of the ith joint angle offset $\Delta \theta_{i}^{0}$, the oscillator input angle $\theta_{i}^{theo}$, the deceleration ratio relative error coefficients $r_{i}$, the return angle $k_{i}$, the direction of return angle compensation $\overset{―}{\theta_{i}^{theo}}$, the coupling coefficient $h_{ij}$ of joint angle. The joint angle $\theta_{i}$ can be expressed as Eq. (5). $h_{ij}$ indicates the coupling coefficient of the angle of articulation $\theta_{j}$ to angle of contact $\theta_{i}$.


$$\theta_{i}=\Delta \theta_{i}^{0}+\theta_{i}^{theo}+r_{i}\cdot \theta_{i}^{theo}+k_{i}\cdot \overset{―}{\theta_{i}^{theo}}+h_{ij}\cdot \theta_{j}^{theo}$$ (5)

Where, $\overset{―}{\theta_{i}^{theo}}$ is determined by the change of the state of motion, can be specifically expressed as Eq. (6).


$$\overset{―}{\theta_{i}^{theo}}=\{\begin{array}{c} {}*20c1,\;Fromreversetoforward\\ \begin{array}{c} -1,\;Fromforwardtoreverse\\ 0,\;else \end{array} \end{array}$$ (6)

The robot positioning error model built from $r_{i}$, $k_{i}$, and $h_{ij}$, can be expressed as Eq. (7).


$$\Delta P_{c}=\frac{\partial P}{\partial \theta_{i}}\theta_{i}^{0}\Delta r_{i}+\frac{\partial P}{\partial \theta_{i}}\overset{―}{\theta_{i}}\Delta k_{i}+\frac{\partial P}{\partial \theta_{i}}\theta_{j}^{theo}\Delta h_{ij}$$ (7)

The robot also generates base mounting errors during the robot mounting process. The error of the base coordinate system $\Delta P_{B}$, can be expressed as Eq. (8).


$$\Delta P_{B}=\frac{\partial P}{\partial r_{0,x}^{B}}\Delta r_{0,x}^{B}+\frac{\partial P}{\partial r_{0,y}^{B}}\Delta r_{0,y}^{B}+\frac{\partial P}{\partial r_{0,z}^{B}}\Delta r_{0,z}^{B}+\frac{\partial P}{\partial p_{0,x}^{B}}\Delta p_{0,x}^{B}+\frac{\partial P}{\partial p_{0,y}^{B}}\Delta p_{0,y}^{B}+\frac{\partial P}{\partial p_{0,z}^{B}}\Delta p_{0,z}^{B}$$ (8)

Where, $r_{0,x}^{B},r_{0,y}^{B},r_{0,z}^{B}$ were the Euler angles of rotation of the base coordinate system, in the X, Y, and Z directions, respectively. $p_{0,x}^{B},p_{0,y}^{B},p_{0,z}^{B}$ were the offsets of the base coordinate system, in the X, Y, and Z directions, respectively.

Synthesizing Eq. (4), Eq. (7), and Eq. (8), the kinematic parameters Δx, to be identified, contains 24 parameters, 6 speed ratio error, 6 return angles, 30 coupling coefficients of joint angles, and 6 parameter errors of the base coordinate system. The robot positioning error model for this paper was jointly built from these 72 parameters.

## 3. Error compensation

### 3.1 The Dog Leg algorithm

**Fig 2:**
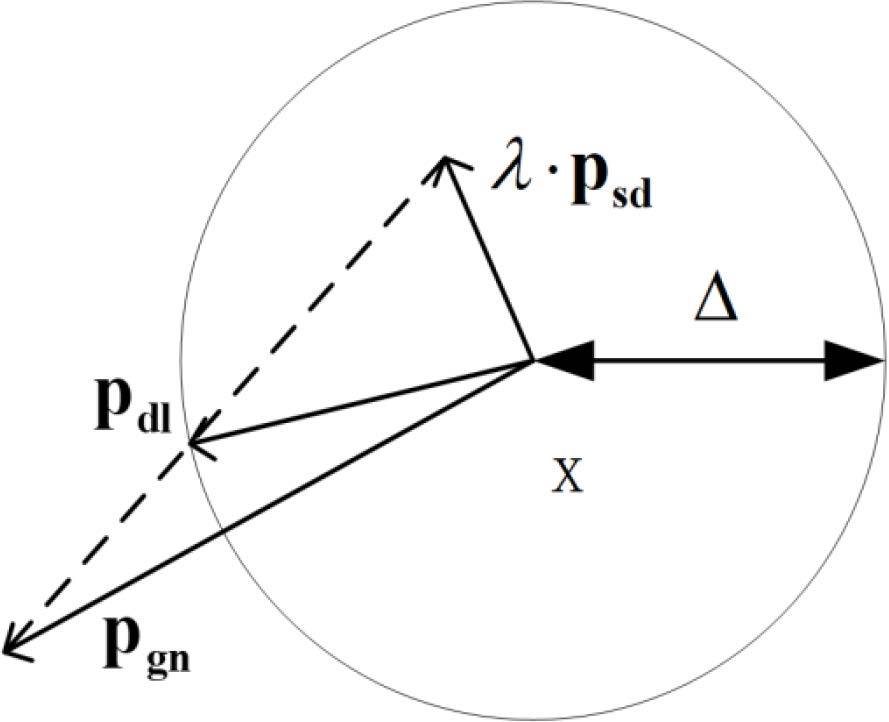
Relationship between the descent direction and trust region of the Dog Leg algorithm. ${𝐩}_{dl}$ indicate the direction of the Dog Leg method. ${𝐩}_{sd}$ indicate the direction of the Fastest Descent method. ${𝐩}_{gn}$ indicate the direction of the Gauss-Newton method. Δ indicate the radius of the Trust Region. λ indicate modulus of ${𝐩}_{sd}$.

In this paper, we propose to calibrate the kinematic parameter, using the Trust Region algorithms. The Dog Leg algorithms is one kind of it, which is combination of the Fastest Descent method and the Gauss-Newton method. The relationship between the direction of the Dog Leg method ${𝐩}_{dl}$, the direction of the Fastest Descent method ${𝐩}_{sd}$, the direction of the Gauss-Newton method ${𝐩}_{gn}$ and the Trust Region Δ was depicted in Fig 2. The Dog Leg algorithm is the enhancement of the Gauss-Newton method. It not only improves the shortcomings, but also improves the global convergence ability [22].

The error e(x) between the theoretical coordinates of the end center and the actual coordinates, measured by the laser tracker. e(x) was used to construct the objective function E(x) of the Dog Leg algorithm, as shown in Eq. (9).


$$E\left(x\right)=\min\left(\frac{1}{2}e{\left(x\right)}^{T}e\left(x\right)\right)$$ (9)

${𝐩}_{sd}$ and ${𝐩}_{gn}$ can be expressed as Eq. (10) and Eq. (11).


$${𝐩}_{sd}=-J^{T}(x)e(x)$$ (10)

$${𝐩}_{gn}=-(J^{T}(x)J(x){)}^{-1}J^{T}(x)e(x)$$ (11)

Where, J(x) is the Jacobi matrix of E(x) to the kinematic parameters. The step size of the most rapid descent method is expressed as Eq. (12).


$$\lambda =-\frac{{{𝐩}_{sd}}^{T}J^{T}(x)e(x)}{{\left\|J(x){𝐩}_{sd}\right\|}_{2}}=\frac{{{𝐩}_{sd}}^{T}{𝐩}_{sd}}{{\left(J(x){𝐩}_{sd}\right)}^{T}J(x){𝐩}_{sd}}$$ (12)

${𝐩}_{dl}$ is determined by Eq. (13).


$$\{\begin{array}{c} {}*20cif\left({\left\|p_{gn}\right\|}_{2}\leq \Delta \right),p_{dl}=p_{gn}\\ elseif\left({\left\|\lambda p_{sd}\right\|}_{2}\geq \Delta \right),p_{dl}=\frac{\Delta }{{\left\|p_{sd}\right\|}_{2}}p_{sd}\\ else,p_{dl}=\lambda p_{sd}+\beta \left(p_{sd}-\lambda p_{sd}\right) \end{array}$$ (13)

Let β satisfies $\left\|p_{dl}\right\|=\Delta$. Let $a=\lambda \cdot p_{sd}$, $b=p_{gn}$, $c=a^{T}\cdot \left(b-a\right)$. β can be expressed as Eq. (14).


$$\beta =\{\begin{array}{c} {}*20c\frac{-c+\sqrt{c^{2}+{\left\|b-a\right\|}_{2}\cdot \left(\Delta^{2}-{\left\|a\right\|}_{2}\right)}}{{\left\|b-a\right\|}_{2}},c\leq 0\\ \frac{\left(\Delta^{2}-{\left\|a\right\|}_{2}\right)}{c+\sqrt{c^{2}+{\left\|b-a\right\|}_{2}\cdot \left(\Delta^{2}-{\left\|a\right\|}_{2}\right)}},c>0 \end{array}$$ (14)

Where, the gain ratio ρ is determined by Eq. (15).


$$\{\begin{array}{c} {}*20cif\left({\left\|p_{gn}\right\|}_{2}\leq \Delta \right),\rho =\frac{\frac{1}{2}{\left(e\left(x_{k}\right)\right)}^{T}e\left(x_{k}\right)-\frac{1}{2}{\left(e\left(x_{k+1}\right)\right)}^{T}e\left(x_{k+1}\right)}{\frac{1}{2}{\left(e\left(x_{k}\right)\right)}^{T}e\left(x_{k}\right)}\\ elseif\left({\left\|\lambda p_{sd}\right\|}_{2}\geq \Delta \right),\rho =\lambda \frac{\frac{1}{2}{\left(e\left(x_{k}\right)\right)}^{T}e\left(x_{k}\right)-\frac{1}{2}{\left(e\left(x_{k+1}\right)\right)}^{T}e\left(x_{k+1}\right)}{\frac{1}{2}\Delta \cdot \left(2{\left\|\lambda p_{sd}\right\|}_{2}^{2}-\Delta \right)}\\ else,\rho =\frac{\frac{1}{2}{\left(e\left(x_{k}\right)\right)}^{T}e\left(x_{k}\right)-\frac{1}{2}{\left(e\left(x_{k+1}\right)\right)}^{T}e\left(x_{k+1}\right)}{\frac{1}{2}\lambda \cdot {\left(1-\beta \right)}^{2}\cdot {\left\|p_{sd}\right\|}_{2}^{2}+\beta \cdot \left(2-\beta \right)\cdot \frac{1}{2}{\left(e\left(x_{k}\right)\right)}^{T}e\left(x_{k}\right)} \end{array}$$ (15)

The trend of Δ is determined by ρ, and the relationship between ρ and Δ can be expressed as Eq. (16).


$$\Delta =\{\begin{array}{c} {}*20c\max\left\{\Delta ,3{\left\|p_{dl}\right\|}_{2}\right\},\rho >0.75\\ \frac{\Delta }{2},\rho <0.25 \end{array}$$ (16)

When ρ > 0, $x_{new}=x_{old}+{𝐩}_{dl}$.

The above process is the basic flow of the Dog Leg algorithm.

The computational cost of the Dog Leg algorithm is very high (about several hours), and this computational cost is not suitable for real-time computation for the time being. Solving the Jacobi matrix is too much time consuming. This limits the practical application of the algorithm.

### 3.2 Error prediction based on the PSONN algorithm

When the predicted point is odd in the workspace of the robot, its error information is missing. At this time, the positioning accuracy is poorly calculated, and the implementation of the algorithm become complication, caused of missing points, and is not general.

**Fig 3:**
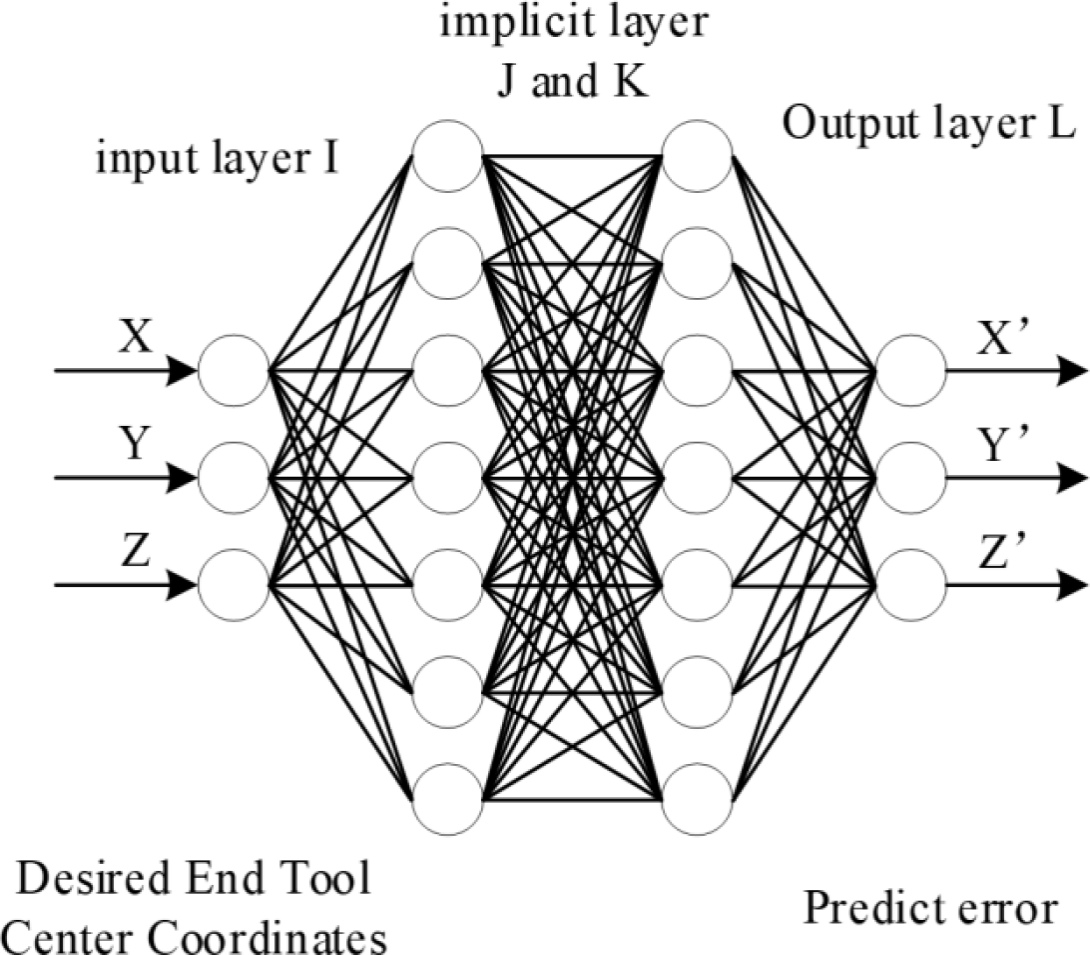
Flowchart of the Neural Network algorithm. I indicate input layer. J and K indicate implicit layer. L indicate Output layer. (X, Y, Z) indicate Desired End Tool Center Coordinates. (X’, Y’, Z’) indicate Predict error of the Neural Network algorithm.

The Neural Network algorithm as nonlinear algorithm can solve the odd problem well. The basic idea of the Neural Network algorithm, is to select the theoretical coordinates of the training samples as inputs, and select the actual coordinates as outputs, to train the Neural Network structure, in order to simulate the error distribution pattern in the robot workspace. Fig 3 shows the flowchart of the Neural Network algorithm in this paper.

The literature [11] demonstrate that the initial weights and thresholds of the Neural Network affect the training accuracy of the Neural Network. The PSO algorithm originates from the bird flock foraging. The approach of each bird foraging is to find out the current location of the bird closest to the food. The PSO algorithm takes advantage of this information sharing system. It allows individuals in the flock to learn from each other. Thus, it can contribute the evolution of the entire flock’s behavior.

First, the position, the velocity, and the fitness of each particle are initialized. The position of the ith particle is denoted as $X_{i}=\left(X_{i1},X_{i2},\cdots ,X_{iM}\right)$, and the velocity is denoted as $V_{i}=\left(V_{i1},V_{i2},\cdots ,V_{iM}\right)$. Where, *i* = 1, 2,...n, n is the number of particles, and M is the dimension. The iteration of the particle position is updated by the iteration of the individual extremes and the population extremes. The particle position of the (*t* + 1) th iteration can be express as Eq. (17). The particle velocity of the (*t* + 1) *t*h iteration can be express as Eq. (18).


$$X_{i}\left(t+1\right)=X_{i}\left(t\right)+V_{i}\left(t+1\right)$$ (17)

$$V_{i}\left(t+1\right)=\omega V_{i}\left(t\right)+c_{1}r_{1}\left(t\right)\left[B_{i}\left(t\right)-X_{i}\left(t\right)\right]+c_{2}r_{2}\left(t\right)\left[B\left(t\right)-X_{i}\left(t\right)\right]$$ (18)

**Fig 4:**
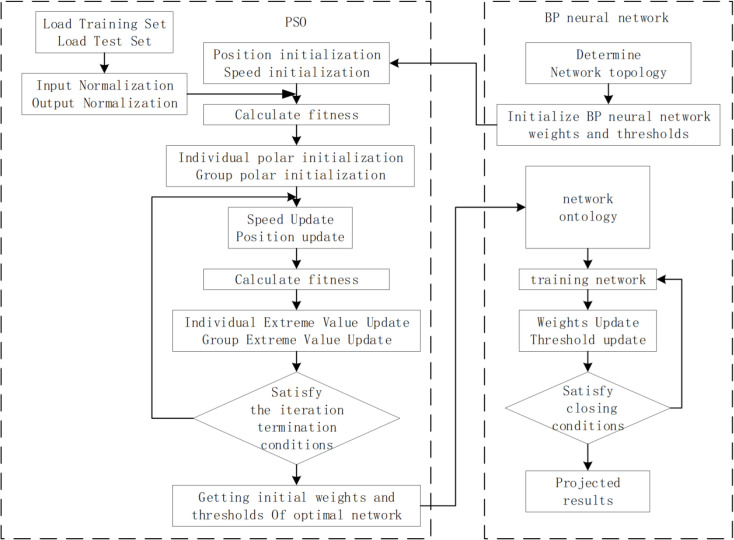
Flow chart of the PSO optimized the Neural Network algorithm. The dashed rectangular frame portion on the left is the PSO algorithm. The PSO algorithm provide initial weights and thresholds of the Neural Network. The dashed rectangular frame portion on the right is the Neural Network algorithm.

Where, $c_{1}$ and $c_{2}$ are the individual acceleration constants and the group acceleration constants, on [0,2], respectively. They regulate the individual and the group approximation to the individual optimum and the group optimum; $r_{1}$, $r_{2}$ are mutually independent stochastic functions, obeying a uniform distribution on [0,1]; ω is the momentum coefficient, non-negative; B(t)$\left(B_{1},B_{2},\cdots ,B_{M}\right)$ is the group optimal extreme value; $B_{i}\left(t\right)$ is the individual optimal extreme value. Fig 4 is the flow chart of the PSONN algorithm proposed in this paper, its initial weights and thresholds of the Neural Network are provided by the PSO algorithm, to prevent the solution of the Neural Network algorithm from falling into the local optimum.

### 3.3 Error interpolation compensation based on the SGMI algorithm

**Fig 5:**
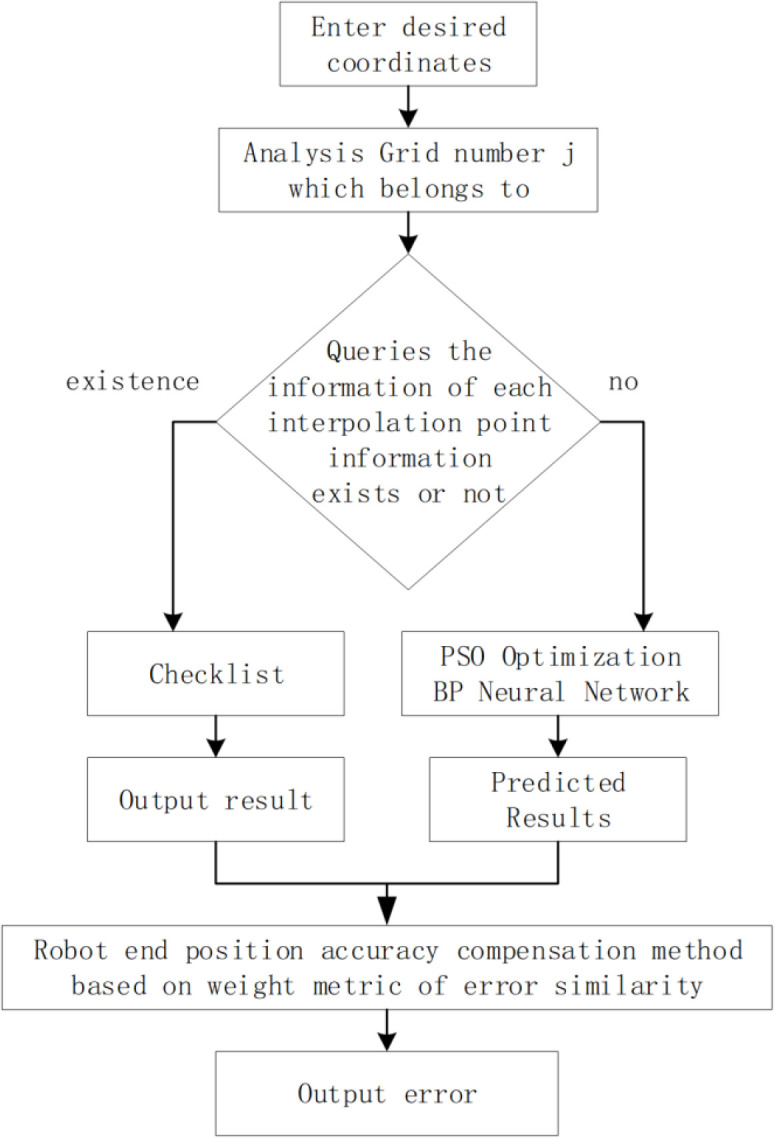
The flow of error interpolation compensation based on the SGMI algorith. Input the desired coordinates, the grid number j is obtained, through the table checklist. If the interpolated point does not exist, the trained network is used to predict the error of the missing interpolated point. At the end, the SGMI algorithm in this subsection is used for prediction.

After calibrating the parameters of the positioning error model, the SGMI algorithm is used to compensate the error of the predicted points. The flow of error interpolation compensation, based on the SGMI algorithm, proposed in this paper, is shown in Fig 5. After inputting the desired coordinates, the grid number is obtain, through the table lookup method, and the information of each interpolation point is extracted. If the error information of the interpolated point does not exist, the trained network is used to predict the error of the missing interpolated point. If the error information of the interpolated point exists, the SGMI algorithm in this subsection is used for prediction.

The basic idea of the SGMI algorithm, is to calibrate the error of each interpolation point of the workspace grid, and then, through the interpolation method, to calculate the prediction point error. When two sets of positions in the Cartesian coordinate system are close, their positioning errors show correlation. The smaller the distance, the higher the correlation. Therefore, the known positioning errors of points in the grid, can be used to interpolate the positioning errors of unknown points, i.e., the inverse of the distance is used as the weight to interpolate the desired point errors.

**Fig 6:**
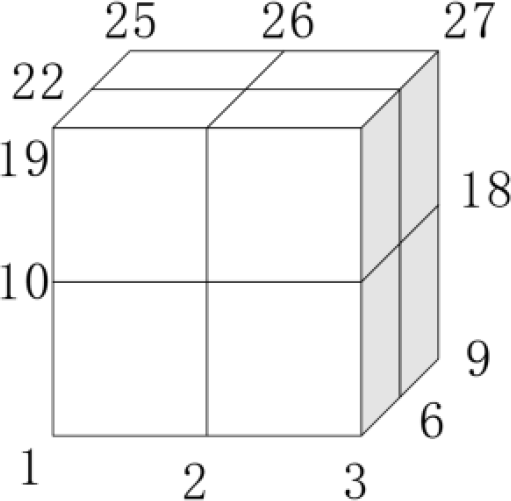
Schematic diagram of spatial grid. The cube grid has 27 interpolation points in total.


As shown in Fig 6, the cube grid has multiple interpolation points $P_{i}$, i=1,2,⋯,n, including 6 face center points, 8 vertices, and 12 line center points. The coordinates of desired point P$\left(x_{i},y_{i},z_{i}\right)$ in the cube grid, the actual coordinates $\left(x_{i}\prime ,y_{i}\prime ,z_{i}\prime \right)$, and the weights of the vertices to the point *P*, are expressed as Eq. (19). Its error (Δx,Δy,Δz), are expressed as Eq. (20).

qi=1/1di\nulldelimiterspacedi∑i=1n1/1di\nulldelimiterspacedi (19)

$$\Delta x=\sum_{i=1}^{n}\Delta x_{i}q_{i},\Delta y=\sum_{i=1}^{n}\Delta y_{i}q_{i},\Delta z=\sum_{i=1}^{n}\Delta z_{i}q_{i}$$ (20)

Where is the distance $d_{i}$ between the desired point and each interpolated points.

## 4. Measurement experiments and results

**Fig 7:**
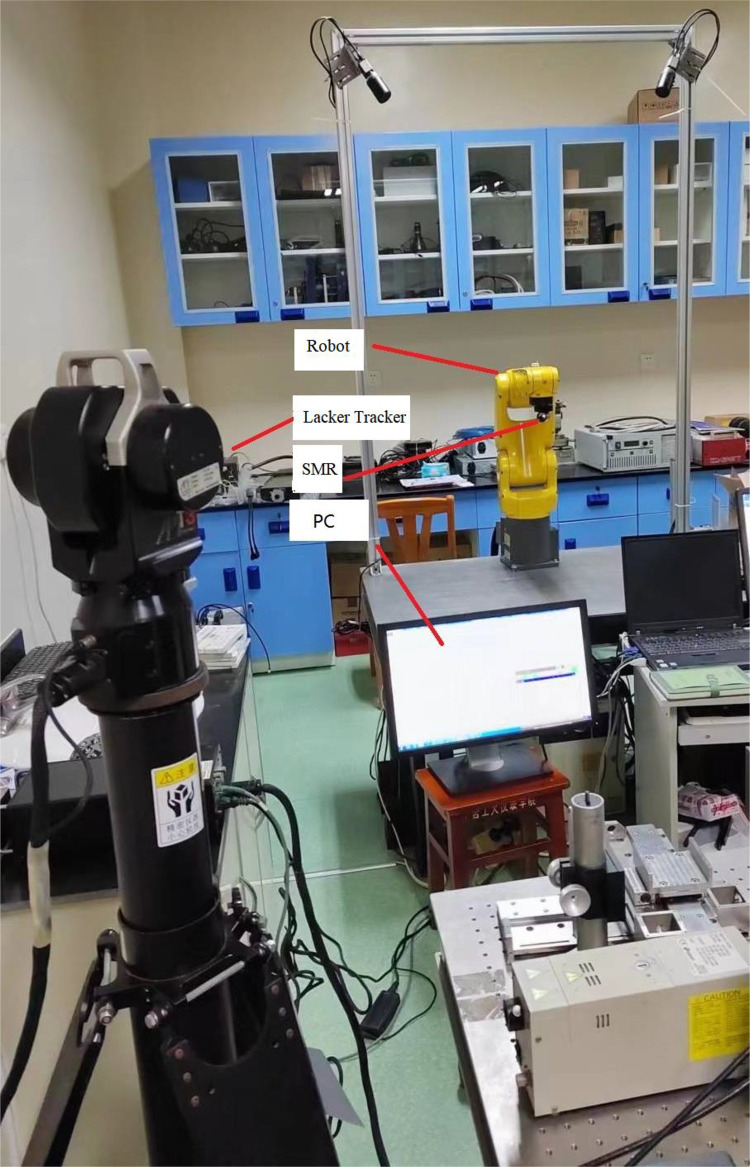
Layout of robot end position measurement. We use the American API T3 laser tracker as calibration tool. SMR (Spherically Mounted Retro) was placed on the end of a six degrees of freedom Robot-FANUC LR MATE.

To validate the comprehensive compensation method for positioning error, proposed in this paper, it was tested on a six degrees of freedom Robot-FANUC LR MATE. The robot end position measurement layout is shown in Fig 7. The robot has repeatable positioning accuracy of 20 μm and joint angle accuracy of 0.01°. We use the American API T3 laser tracker as calibration tool. API T3 has measurement accuracy of 15 μm. SMR (Spherically Mounted Retro) was placed on the end of the robot. The measurement points were planned, so that they were discretely and uniformly distributed in the robot workspace. Total of 863 points were measured, utilizing this laser tracker.

The transformation matrix $T_{7}^{M}$ from the laser tracker coordinate system to the center of the robot end, is expressed as $T_{7}^{M}=T_{B}^{M}\left(T_{1}^{0}T_{2}^{1}T_{3}^{2}RT_{4}^{3}T_{5}^{4}T_{6}^{5}\right)T_{7}^{6}$.

Before the kinematic parameter identification, there are two sets of coordinate system transformations that are unknown, i.e., $T_{B}^{M}$ and $T_{7}^{6}$. $T_{B}^{M}$ was the transformation of the laser tracker coordinate system to the base coordinate system. $T_{7}^{6}$ was the transformation of the end coordinate system to the SMR coordinate systems. They can be computed by the following process [23] $T_{7}^{6}=\left[\begin{array}{cccc} {}*20c1 & 0 & 0 & -30.53\\ 0 & 1 & 0 & -11.423\\ 0 & 0 & 1 & 11.067\\ 0 & 0 & 0 & 1 \end{array}\right]$$T_{B}^{M}=\left[\begin{array}{cccc} {}*20c-0.906 & 0.422 & -0.007 & 2264.481\\ 0.422 & 0.906 & -0.010 & 154.016\\ -0.002 & 0.013 & 0.999 & -323.252\\ 0 & 0 & 0 & 1 \end{array}\right]$.

### 4.1 Kinematic parameter identification experiment

**Fig 8:**
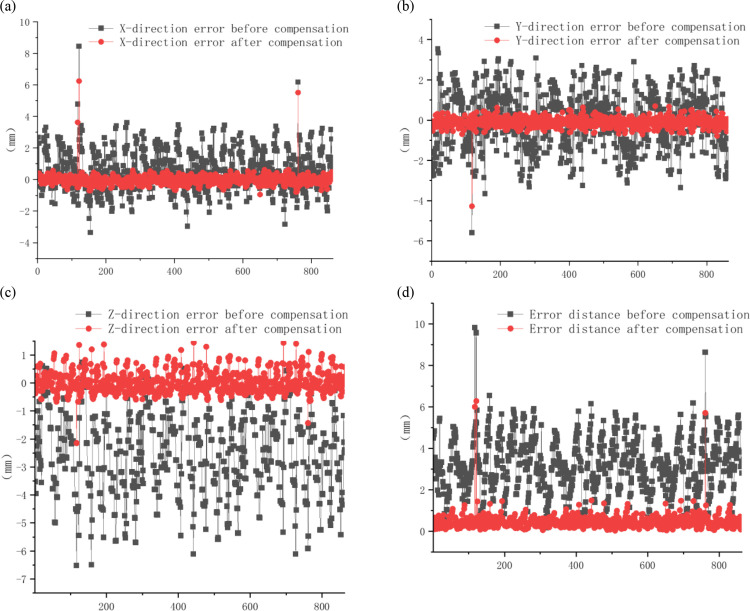
Comparison of error before and after parameter compensation. (a) Position error in the X direction before (black) and after (red) parameter compensation. (b) Position error in the Y direction before (black) and after (red) parameter compensation. (c) Position error in the Z direction before (black) and after (red) parameter compensation. (d) Error distance before (black) and after (red) parameter compensation.

Based on the pre-identification, 72 kinematic parameters are identified, using the Dog Leg algorithm. The robot position error before and after compensation, is shown in Fig 8. The maximum error, the average error, and the variance of the robot end in three directions and the error distance, before and after compensation, are shown in Table 2.

**Table 2:** Comparison of position errors before and after kinematic parameter compensation (mm).

		Before compensation	After compensating 24 kinematic parameters	After compensating 72 parameters
X	minimum error	−3.363	−0.944	−0.953
	maximum error	8.448	6.247	5.833
	average error	0.533	−0.034	−0.006
	variance	1.519	0.159	0.137
Y	minimum error	−5.592	−4.277	−4.140
	maximum error	3.532	0.697	0.800
	average error	−0.041	−0.098	0.010
	variance	1.800	0.069	0.090
Z	minimum error	−6.516	−2.146	−1.937
	maximum error	0.732	1.443	0.672
	average error	−2.455	0.003	−0.060
	variance	2.073	0.145	0.066
D	minimum error	0.173	0.032	0.035
	maximum error	9.828	6.283	5.993
	average error	3.158	0.473	0.406
	variance	1.726	0.160	0.132

After compensating 24 basic kinematic parameters, the average position error decreases from 3.158 mm to 0.473 mm. The maximum position error decreases from 9.828 mm to 6.283 mm. The average position error in X direction decreases from (0.533) mm to (−0.034) mm. The average position error in Y direction increases from (−0.041) mm to (−0.098) mm. The average position error in Z direction decreases from (−2.455) mm to (0.003) mm. After compensating total 72 parameters, the average position error is 0.406 mm. Solving it several times, it can get the same result, which shows that the stability and reliability of the algorithm proposed in this paper is better.

A total of 100 random points were collected using Laser Tracker to compare the traditional FD, GN and Dog Leg algorithms, with each other. The FD algorithm do not converge. The results of the simulation experiments are listed in Table 3. The robot positioning error is reduced from 3.091 mm to 0.551 mm and 0.550 mm after calibration of GN and DG algorithms, respectively, and the standard deviation is reduced from 1.789 mm to 0.081 mm, and 0.032 mm, $\theta_{2}^{0},\;\theta_{3}^{0},\;\alpha_{0},\;\alpha_{3},\;\theta_{1}^{0},\;\alpha_{1}$. The angular parameters in the kinematic model have a greater effect on the error than the length parameters, and the joint parameters of more closed to base, have a greater effect on the error than the terminal joint parameters.

**Table 3:** Comparison of position errors before and after calibration.

Errors (mm)	Before calibration	After FD	After GN	After DG
Max	6.155	not converge	1.407	0.981
Mean	3.091		0.551	0.550
STD	1.789		0.081	0.032

### 4.2 Experiments on error prediction based on the PSONN algorithm

After many experiments, it is determined that the Neural Network algorithm, includes the input layer with 3 nodes, the hidden layer J with 7 nodes, the hidden layer K with 7 nodes, and the output layer with 3 nodes. The training function of the Neural Network algorithm is ‘train LM’; the learning rate is 0.1; the total number of samples is 1331; the training number of samples is 1200; the validation number of samples is 131; the number of weights is 112; and the number of thresholds is 20; The 3 input nodes are desired coordinates; the 3 output nodes are localization coordinates.

After many experiments, it is determined that the PS0 algorithm, with the number of populations as 50, the particle dimension as 112, the number of evolutions as 600, the maximum value of individual velocity as 1, the minimum value of individual velocity as −1, the maximum value of individual factor as 200; the minimum value of individual factor as −200, the maximum values of optimal acceleration for the individual and the population as 1, the minimum values of optimal acceleration for the individual and the population as −1.

To verify the accuracy of the proposed PSONN algorithm, the prediction results are compared with the traditional vertex interpolation method. In the robotic workspace, 1000 grids were divided.

The average prediction accuracies of the grid center point in the X, Y and Z directions and the error distance are approximately 0.0531 mm, 0.0491 mm, 0.1516 mm and 0.1721 mm, respectively. Although it does not reach the prediction accuracy level of the classical 8 points interpolation method, i.e., 0.0593 mm, it basically reaches the current level of robot repetitive positioning accuracy, around 0.15 mm.

The average prediction accuracies of the 1000 grid centroids in the X, Y, and Z directions are 0.0531 mm, 0.0491 mm, and 0.1516 mm, and the error distances are approximately 0.1721 mm, which is less than that of the classical 8-point interpolation method. The error distance is approximately 0.1721 mm, which is higher than 0.0593 mm of the classical 8-point interpolation method, but basically reaches the nominal repeatable positioning accuracy level of 0.15 mm of the robot.

### 4.3 Robot end positioning error interpolation compensation

Based on the number of interpolation points in the grid, that participate in the compensation calculation, these cases are defined as 6-point interpolation (face center point), 8-point interpolation (grid vertices), 12-point interpolation (line center point), 14-point interpolation (face center point, grid vertices), 18-point interpolation (face center point, line center point), 20-point interpolation (grid vertices, line center point), 26-point interpolation (face center point, grid vertices, line center point).

**Fig 9:**
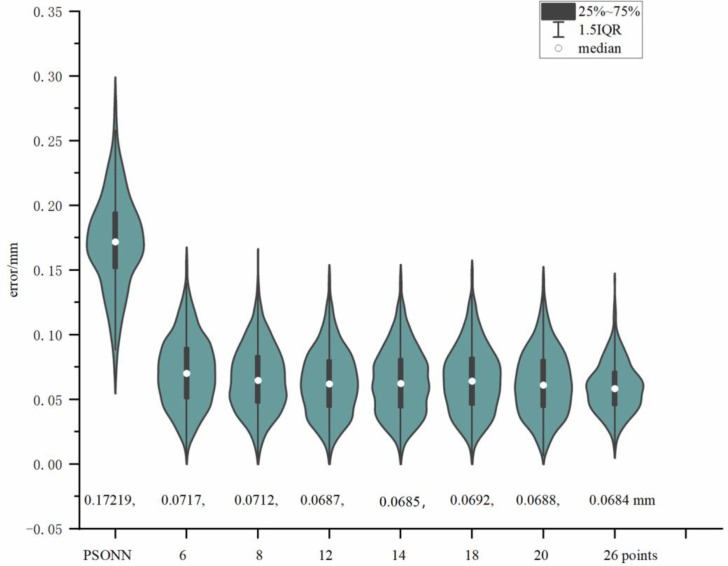
Comparison of multipoint interpolation method. We propose 6 kinds of multipoint interpolation. The average error of 6-point interpolation (face center point) is 0.0717 mm. The average error of 12-point interpolation (line center point) is 0.0687 mm. The average error of 14-point interpolation (face center point, grid vertices) is 0.0685 mm. The average error of 18-point interpolation (face center point, line center point) is 0.0692 mm. The average error of 20-point interpolation (grid vertices, line center point) is 0.0688 mm. The average error of 26-point interpolation (face center point, grid vertices, line center point) is 0.0684 mm. Those 6 kinds of multipoint interpolation and the SGMI algorithm are compared with 8-point interpolation with the average error of 0.0712 mm.

These 6 kinds of multipoint interpolation are compared with 8-point interpolation, and the result is demonstrated in Fig 9. 14-point interpolation has the highest accuracy, and it is better than the classical 8-point interpolation and other multipoint interpolation methods.

## 5. Conclusion

This paper proposes a new algorithm to reduce the robot error in progressive steps, which combines the interpretability of the traditional algorithm and the nonlinear effect of the neural network, and avoids the low precision of the traditional algorithm and the local optimal phenomenon of the neural network. This paper establishes a positioning error model, for industrial robot FANUC LR MATE with six degrees of freedom, which contains kinematic parameter, return angles, deceleration ratio relative error coefficients, joint angle coupling coefficients, and base coordinate system errors. Firstly, the unknown parameters in the robot positioning error model are identified, based on the enhanced Dog Leg algorithm, in two steps. The correctness of this method is verified by experiments. The experimental results demonstrate that, the first step, is to calibrate 24 basic kinematic parameters, the robot end positioning error is reduced from approximately 3.158 mm to approximately 0.473 mm; the second step, is to calibrate all 72 parameters, the robot positioning error is reduced from approximately 0.473 mm to approximately 0.406 mm. Secondly, to obtain the error of the odd point in the robot workspace, the PSONN algorithm is proposed. The experimental results demonstrate that although the prediction accuracy of the PSONN algorithm is approximately 0.1721 mm, which is inferior to the accuracy level of the grid multipoint interpolation method, i.e., approximately 0.0593 mm, it can make up for the missing singularity error of the grid multipoint interpolation well. Thirdly, the SGMI algorithm is proposed to compensate the error. The 6-point, 12-point, 14-point, 18-point, 20-point and 26-point interpolation methods are compared with the classical 8-point interpolation method. The experimental results demonstrate that the 14-point interpolation method has the best error compensation effect and can reduce the robot positioning error from approximately 0.406 mm to approximately 0.0685 mm.
